# Supplementary figures and images for: Acute net stress of young adult zebrafish (Danio rerio) is not sufficient to increase anxiety-like behavior and whole-body cortisol
Source: PeerJ. 2019 Aug 7;7:e7469. doi: 10.7717/peerj.7469 (PMC6689218; doi:10.7717/peerj.7469)

**A**

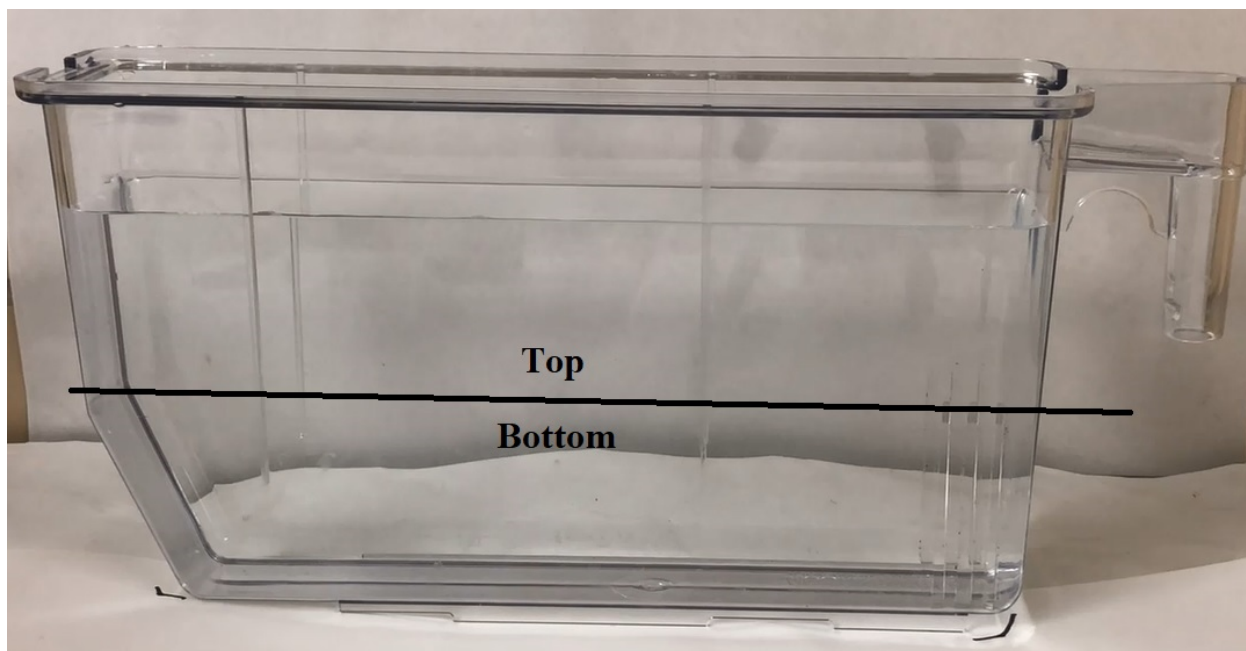

**B**

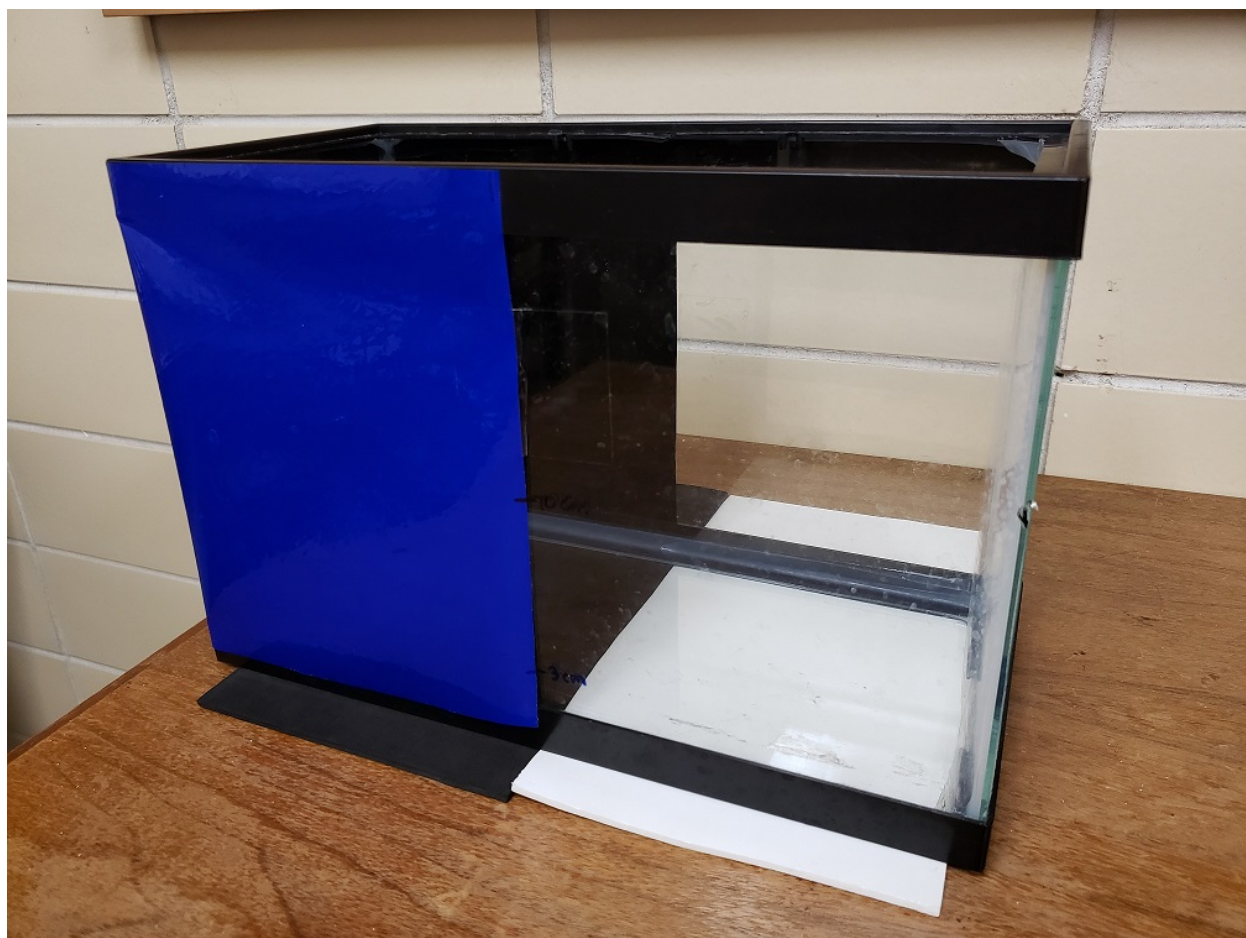

Supplement: Figure S1 — The first picture (A) indicates the novel tank used in Experiment 1 and the second picture (B) indicates the tank used in Experiment 2. [file peerj-07-7469-s002.pdf]
